# The Units of General Hospital Construction

**Published:** 1907-06-15

**Authors:** 


### HOSPITAL ADMINISTRATION.

### CONSTRUCTION AND ECONOMICS.

### THE UNITS OF GENERAL HOSPITAL CONSTRUCTION.

#### THE MEDICAL OFFICER AND THE WARD UNIT.

Some criticism has arisen on previous articles in this section owing to the fact that in the medical and surgical ward units, a flat has been provided for the resident medical officer in charge. This is the Scotch system of hospital construction and this week we propose to set forth the reasons which may be urged for and against the universal adoption of this plan, in modern hospital construction.

It will be observed that in the plan of a complete ward unit, illustrated in a previous article, accommodation is provided therein for a resident medical officer. Such a departure from the older method of housing the resident medical staff, where centralisation was the rule, may require some explanation and justification. But the advantages so completely outweigh the disadvantages that this provision for a resident in each unit will no doubt eventually become universal wherever the unit system is adopted.

The rapid progress of bacteriology, hæmatology, electro-therapeutics, not to mention the developments in surgery, foster the ever-increasing tendency to specialise; and for young men who wish to devote themselves to original work in any special department there is no better opportunity than a resident post in one of our modern hospitals. There he has the clinical material. There the most elaborate plant and equipment, with every facility for study and research, are at his disposal, with an increasing number of post-graduate fellowships, lectureships, and other opportunities for those who wish to pursue any particular line of study. The inevitable result is that in a large up-to-date general hospital the post of such a resident is an envied one, and commands, as a rule, the most distinguished graduates of their time. These graduates seldom receive a salary, or at most a purely nominal one, although the amount of work which they accomplish for the institution represents a considerable asset to it. Their main desire in so attaching themselves to a hospital is to gain as much knowledge and experience as possible and to profit by the special facilities it may afford for some particularly congenial work.

The hospital, then, secures the willing and valuable service of its resident medical officers practically without outlay beyond their board and residence. It is but reasonable that, in return, the provision for their comfort and convenience should be the best available. A man cannot do original or satisfactory work or prepare a thesis, say for a higher degree, if he shares his rooms with another with whom, it may be, he has but little in common. The man who wishes the necessary quiet for study cannot obtain it with a partner whose pet hobby is Wagner or Beethoven and who is at the piano in their sitting-room or the room adjoining at every conceivable opportunity. This is no fancied picture, but an actual experience.

To the resident, therefore, the advantage in having his rooms in his ward unit is obvious. Here he has the strictest privacy. He has facilities for work when he is not required in his wards. There is no undesirable intrusion or unwelcome interruption to his studies. It is to all intents and purposes his little home. He can see his friends in his own sitting-room, and when he wishes the company of his *confrères* he can obtain this either in the common dining-room or billiard-room, or by making his own arrangements for visitation. This is entirely in his own hands and under his own control.

But the advantage is not only on the side of the resident. The benefit to the hospital is even greater. The main object in having a medical officer in residence is that the services of a qualified medical man, familiar with the detail of each case, may be available on the shortest notice. It is quite conceivable that many cases, such, for example, as hæmorrhage, uræmia, tetanus, fits, and others that readily occur to one, may require the immediate attention of the medical officer at any hour of the night or day, and that the notice may be of the very shortest. Where the resident's quarters are within his unit he is always on the spot when he has a critical case under his care, and yet need not hang about his wards awaiting developments. Under the old arrangement whereby the residents resided in some central block, the time occupied by the nurse in getting into communication, either by telephone or messenger, and that required for the recipient to reach the bedside, all meant dangerous delay. Under the old system no resident medical officer can justly be held responsible for such delays.

It may be claimed that planning the residents' quarters in each unit involves extra service, and therefore increased cost and greater difficulty in administration. The quarters of the medical officer, although forming part of the unit, are not under charge of the ward sister. The care of them devolves on the housekeeper, as the domestic arrangements in well-administered hospitals are quite distinct from the curative. The domestic staff is under the supervision of a housekeeper, and the residents' quarters, no matter where situated, are under her immediate care. The more scattered these quarters, therefore, the more inconvenient will be their supervision. This disadvantage, however, is more theoretical than real, and what actually exists is readily overcome. The distances to be covered are no greater for the housekeeper than for the superintendent and matron, who have to supervise every ward, and with the convenience of lifts to each floor this is reduced to a minimum. The supply of coal for his rooms is delivered by the porter when he delivers that for the ward, kitchen, etc.

Then it must be clearly understood that the resident staff have no food in their private apartments. The mess-room, billiard-room, and smoking-room are common rooms; and even in case of illness it is found much more satisfactory to transfer the invalid to one of the side wards where he can be properly treated and nursed, leaving his apartments free for the substitute who undertakes his duties. So that under no circumstances is food served in his quarters. The general mess-room is situated in the central administrative building convenient to the kitchen and service rooms, and should, of course, be in telephonic communication with all the ward units.

It must not be supposed that a housemaid is required in each unit to attend to the resident's rooms. In a well-appointed hospital where lifts lead to each floor one maid to every five suites of rooms is all that is requisite to overtake the work and do it thoroughly. The inconvenience to the supervising housekeeper is trifling, and any additional labour involved as compared with the older method is so infinitesimal that there is no appreciable or calculable increase in the cost of administration. The increased efficiency obtained more than counterbalances any question of inconvenience.

The quarters assigned to the resident medical officer in the ward unit plans already shown consist of a sitting-room, bedroom, and bathroom. These are entered from a private vestibule, so that there is complete privacy; and the experience so frequently met with under the old system of several residents having to wait their turn for a bath is wholly avoided. Each resident has his complete quarters in his unit. His unit is more under his direct supervision, and his status in the hospital is correspondingly increased. Nowhere is the advantage of this arrangement better exemplified than in the admission department. Regrettable delays occasionally occur where the quarters of the casualty medical officer are situated at some distance from his department, and there is no justification for perpetuating a system of hospital construction and administration, which renders such avoidable delays possible, when it can be demonstrated that the complete unit system with the residents' quarters incorporated does not materially add to the cost of working.

